# Health schools as an organizational form of realization of the “life course health development” concept

**DOI:** 10.25122/jml-2021-1127

**Published:** 2021

**Authors:** Anna Fomina, Lyudmila Maksimenko, Evgeniya Atsel

**Affiliations:** 1. Department of Public Health, Healthcare and Hygene, Peoples' Friendship University of Russia (RUDN University), Moscow, Russian Federation; 2. Kazan State Medical Academy, Branch of the Russian Ministry of Health of the Russian Federation, Kazan, Russian Federation

**Keywords:** life course health development, Tatarstan, health schools, cardiovascular diseases, mortality

## Abstract

This study was conducted to evaluate the effectiveness of health education in the Tatarstan Republic by establishing educational programs – Health Schools – for groups of patients with a high risk of developing potentially fatal cardiac and respiratory conditions. The concept of “Life Course Health Development” implies the development of mechanisms for personalized health management. The goal of the study is to explore the effectiveness of the specialized Health Schools in Tatarstan. For the comparative study of health education effects on the overall state of personal health, 590 patients were surveyed in a randomized controlled trial. The groups of patients were compared in relation to their health education; their health status was observed prior to and afterward undergoing the educative preventative programs and estimated in comparison between the two groups. Extrapolation of the data on Tatarstan’s patient population was obtained through this study, taking into account the state of health of the Health Schools students, obtaining the regression equations of population mortality and the effects of training on it. The effectiveness of Health Schools for patients with cardiovascular pathology has been proven. However, additional efforts are required to involve a wider range of patients and increase learning effectiveness to critical levels of awareness by introducing new forms of education in Health Schools since it statistically significantly increased the awareness level regarding disease nature and preventive measures.

## INTRODUCTION

Today, a healthy lifestyle is widely considered the only economically feasible and productively perceptive way of developing healthcare systems [1]. “Life Course Health Development” (LCHD) is a fundamentally new approach to a healthy lifestyle developed by Neal Halfon, the Director of the UCLA Center for Healthier Children, Families, and Communities (Los Angeles, United States of America) and his colleagues; this approach was intensively explored and improved for the past 20 years concerning its improvability on healthy development as a complex dynamic process starting before conception and lasting for the entire lifetime, guiding the development of new strategies aimed at optimizing the health paths of individuals and the population [2–4]. Health Schools for patients with cardiovascular diseases in Russia – a condition which happens to be the significant factor in mortality among the adult population – implemented this approach.

According to LCHD, healthy lifestyle training is essential for the healthy development of people who already had developed noncontagious health conditions or metabolism disorders. The World Health Organization recommends providing health education to improve the population’s well-being by raising awareness and fixing people’s attitudes towards general fitness.

### Health School structure and objectives

Health School for patients and individuals who are exposed to risk factors is an organizational form of group counseling of preventative nature (health training and education in accordance with ICD-10 class XXL, Z70-76); a preventive medical service rendered to patients or individuals with a high risk of developing cardiac conditions would be a prime staple of healthcare and fitness conditioning. Health Schools are meant to reach objectives as

- improvement of the public’s (patient’s) awareness regarding the disease’s nature and the risk factors associated with it;
- psychological understanding of the responsibility for managing one’s health;
- development of rational and proactive attitude towards the disease;
- motivation for health improvement;
- devotion to treatment and abidance by the doctor’s recommendations;
- formation of skills and abilities to control one’s health state, and rendering first premedical aid in case of disease exacerbations and crises without assistance;
- development of patients’ skills and abilities to mitigate the adverse impact of behavioral risk factors onto one’s health (by optimizing the nutrition and motor activity, breaking bad habits, managing one’s stress).

In practical terms, Health Schools should develop the skills and abilities necessary to analyze unhealthy factors and forming an individual health improvement plan.

### Mortality rates and risk factors

Circulatory system diseases (ICD-10, I00-I99) are the main cause of mortality around the globe, reaching a number of 17 million fatalities per year. The prevalence of obesity, diabetes, dyslipidemia and hypertension combined with metabolic syndrome leads to increased cardiovascular disease incidence. According to the Global Burden of Diseases (GBD) database compiled by The Institute for Health Metrics and Evaluation (IHME), cardiovascular diseases are the long-standing leader among the mortality factors in Russia [5].

Among the cardiovascular disease risk factors by the index of years lost due to disability (YLD), specifically for the Russian population, IHME distinguishes high body weight index, alcohol consumption, high systolic blood pressure, high fasting blood plasma glucose, and smoking, which are fully or partially due to the lifestyle and, consequently, attributed to the group of modifiable risk factors. Kones emphasized that coronary artery disease can be prevented with relatively simple and inexpensive lifestyle changes, which are the essential measure of public healthcare [6]. According to the LHCD concept, individuals in the risk groups should be a special object of precision (patient-specific) medicine and healthcare.

The Healthcare Access and Quality (HAQ) index was introduced in 2015 to assess patient-specific healthcare performance [7]. A scale rated from 1 to 100 is used for the means of measurement of that index. The average HAQ index for Russia is equal to 72 (Panel of countries A). However, for some individual cardiovascular diseases, the value of the HAQ index is far below average, equaling 63 for hypertensive heart diseases, 41 for cerebrovascular diseases, and 36 for coronary heart disease. In Russia, The HAQ index dynamics expressed as the average difference between the highest achieved and all of the observed levels shows that this difference was scaling between 17 and 21 in 1990 and between 13 and 17 in 2016, indicating positive changes.

According to the “Healthcare in Russia” yearly statistical report, the incidence of circulatory system diseases in 2016 reached 23,617.5 per 100,000, making it the second most prevalent cause of mortality after respiratory diseases (29.8%). The mortality rate for the active populous of the working-age reaches 156.7 per 100,000. In the Republic of Tatarstan, the share of circulatory system diseases in 2016 reached 14.08% of the total incidence, 3.88% of the primary diseases incidence, 6.92% of the incidence resulting in short-term disability (by the number of cases), 29.42% of permanent disability cases, and 51.72% of deaths among all disease categories. A large part of circulatory diseases includes ischemic heart disease (17.6% of the prevalence, 19.16% of the incidence of primary diseases, 12.83% of the incidence followed by short-term disability (by the number of cases), 39.93% of the disability rate, 39.37% of the mortality rate from diseases of the circulatory system) and diseases characterized by high blood pressure (40.67% of the prevalence, 27.6% of the incidence of primary diseases, 6.42% of the disability rate, 6.54% of the mortality rate due to circulatory system diseases). As a whole, the trending numbers of circulatory system conditions and related to fatalities in the Republic of Tatarstan seem to be decreasing.

By enlarge, the international healthcare community emphasizes the importance of health education of the population and patients in their search for optimal cardiovascular disease control solutions. Heart failure (HF) is the primary burden of healthcare, and the demand for the development of health support and quality of life strategies for people with heart failure increases permanently [8]. According to Aspromonte *et al*., “The shift from acute to chronic care mandates a proactive role of healthcare professionals in building efficient network system and chaperone HF patients through them” [9].

### Effectiveness of Health Schools and International Implementations of similar programs

The effectiveness of Health Schools for patients with cardiovascular diseases, hypertension, and type 2 diabetes mellitus has been acknowledged by researchers from Saudi Arabia, South Korea, and other countries [10–13]. The first Korean guidelines on chronic heart failure (CHF) from 2017 emphasized the importance of education and counseling on nutrition, weight control, smoking, and alcohol consumption, vaccination and prevention of infective endocarditis, administration of drugs and dietary supplements, work and life activities in general, including sexual activity, domestic physical exertion, flying on airplanes, methods of rehabilitation and physiotherapy, and management of concomitant diseases [14]. In the review of published data on the nutrition of CHF patients (n=1045, 2005–2015), Abshire *et al*. provided educational and prescriptive recommendations and created a criteria base for developing therapeutic nutrition programs for CHF patients [15]. A meta-analysis of 51 reviews on health education of patients with a high risk of acute HF (1,324 studies, 288,057 patients) showed the effectiveness of health education [16]. The guidelines for managing patients with unstable angina include the National Cholesterol Education Program in the United States of America; such programs are recommended for patients with a 10-year or longer period of developing symptoms of chronic heart disease and being prone to two or more risk factors [17].

Thus, being the root cause of mortality in Russia, cardiovascular diseases are controllable even at their advanced stages and have a high socioeconomic significance for the healthcare system and society. They could be controlled through Health Schools, the need for which is undoubted, provided they effectively achieve the goals and objectives set. The purpose of this research is to determine the effectiveness of training patients with arterial hypertension (AH), stable angina pectoris (SAP), and chronic heart failure (CHF) at the Health Schools from the Republic of Tatarstan.

## MATERIAL AND METHODS

The subject of this randomized control survey was those patients (n=590) who received primary medical care between 2005 and 2015. All study stages were approved by the Ethics Committee of the Kazan State Medical Academy, Branch Campus of the Russian Ministry of Health of the Russian Federation. This sample included 388 patients who constituted the main group of specialized Health School students, including patients with AH (n=347), SAP (n=210), CHF (n=163), and 202 patients who were included in the untrained control group, including patients with AH (n=172), SAP (n=121), and CHF (n=87).

First of all, the awareness range was studied in patients with hematological diseases about procedures and algorithms, their application, and tested connection of pathologies with the influence rate of the diseases to people’s everyday lifestyle. The subjects were under the general practitioners’ observation. The testing was made under special-designed cards:

- the research card of the patient’s awareness range in the prevention of hypertonia disease (arterial hypertonia) during the first visit to the doctor;
- the research card of the patient’s awareness range in the prevention of coronary heart disease (CHD) with SAP during the first visit to the doctor;
- the research card of the patient’s awareness range in the prevention of CHF during the first visit to the doctor.

Different levels of training programs were selected depending on the changes in the patients’ health status. The effectiveness of training at the Health Schools was assessed by the level of awareness of preventing the complications in existing health conditions identified (based on compiled questionnaires containing 20 questions) and represented by the number of correct answers at the beginning of the training period and after its completion. The long-term training effects were estimated by the mortality rate per 100,000 people from cardiopulmonary arrest, acute myocardial infarction, and circulatory diseases within a ten-year period, depending on the effectiveness of individual patient’s training at Health School. Based on this survey, a database containing statistical analysis was developed using the Statistical Package for the Social Sciences (SPSS) software. This data was analyzed using a t-test, Pearson’s correlation coefficient, and regression analysis within the 95% confidence interval.

## RESULTS

The assessment of the awareness range in the prevention of cardio-hematological diseases showed a low awareness level for both tested groups at the beginning of the observation period. The average awareness range was 11.92 and 11.66 points (the general and control group, respectively) for AH (p>0.05), 13.01 and 13.27 points for SAP (p>0.05), and 12.28 and 12.52 points for CHF (p>0.05). The assessment conducted at the ending of the observation period showed an increase in the average awareness range regarding AH knowledge to 15.13 points (p>0.001). For SAP, this value was 15.64 points (p>0.001), and 14.71 points for CHF (p>0.001).

No statistically significant difference was noted regarding disease prevention awareness between subjects in the general group (trained at the Health School) and subjects in the control group at the beginning of the observation period (p>0.05). In contrast, at the end of the observation period, the differences were distinguishable: p<0.001 for AH patients on all questions related to AH prevention; p<0.05 for SAP patients, except for the answers related to the questions about whether any pain in the heart area was a manifestation of angina and whether it was necessary to limit the content of easily digestible carbohydrates from food (p>0.05); p<0.05 for CHF patients, except for the question about the danger of smoking (p>0.05), which indicates that patients from the control group obtained information on these questions from other sources.

The SAP patients demonstrated the highest initial level of awareness (13.0±0.2 out of 20 points). The CHF patients ranked second (12.3±0.2), and the AH patients ranked third (11.9±0.1). The training raised awareness on disease and prevention with statistical significance (p<0.001), but the progress of the AH patients was the most important (+3.2 points from the initial level). In terms of effectiveness, the training of SAP patients ranked second (+2.6 points), and the training of CHF patients ranked third (+2.4 points) (Table 1).

**Table 1:** Comparison of the average awareness of patients with circulatory system diseases after training at the specialized Health Schools.

Nosological forms of circulatorysystem diseases	Observation points	Average prevention awareness level (out of 20 points)	P-value
**Arterial hypertension**	At the beginning	11.9±0.1	<0.001
	In the end	15.1±0.1	
**Angina pectoris**	At the beginning	13.0±0.2	<0.001
	In the end	15.6±0.2	
**Chronic heart failure**	At the beginning	12.3±0.2	<0.001
	In the end	14.7±0.2	

Assuming the hypothesis that the indicated average levels of awareness on the prevention of circulatory system diseases reflect the awareness of the adult population of Tatarstan and extrapolating the average levels of awareness of patients with circulatory system diseases who attended and those who had not attended the Health Schools are correct, a regression and correlation analysis can be conducted in order to identify the relationship between mortality from the corresponding causes and the average awareness levels depending on Health School attendance.

The correlation between mortality per 100,000 of the adult population in Tatarstan and the average prevention awareness of patients with hypertension, angina pectoris, and chronic heart failure was estimated as moderate according to the Chaddock scale. It had been shown that a decrease by 1 point in the awareness level indicates an increase in the number of deaths by 31.27 among patients with AH, by 7.1 in patients with angina pectoris and by 18.7 in patients with chronic heart failure.

The following equations are the regression equations of mortality due to stroke from the average level of hypertension prevention awareness of patients (1); due to acute myocardial infarction from the average level of SAP prevention awareness of patients (2); due to circulatory system diseases from the average level of CHF prevention awareness of patients (3):


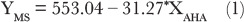


where Y_MAMI_ is the mortality rate of the adult population from acute myocardial infarction (per 100,000 population of the corresponding age), X_APA_ is the average awareness of patients regarding angina pectoris prevention (in points).


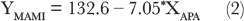


where Y_MAMI_ is the mortality rate of the adult population from acute myocardial infarction (per 100,000 population of the corresponding age), X_APA_ is the average awareness of patients regarding angina pectoris prevention (in points).


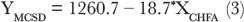


where YMCSD is the mortality rate of the adult population from circulatory system diseases (per 100,000 population of the corresponding age), X_CHFA_ is the average awareness of patients regarding the secondary prevention of chronic heart failure (in points).

The analysis of the regression equations is shown in Table 2. The regression models made it possible to predict mortality rates per 100,000 with zero levels of the patients’ prevention awareness: 553.04 (stroke in AH), 132.6 (acute myocardial infarction in SAP), and 1260.7 (circulatory system diseases in CHF). According to the models, a reduction of fatalities due to stroke in patients with arterial hypertension to zero can be achieved at the critical awareness level of 17.7 points (out of 20) and acute myocardial infarction in SAP patients at the level of 18.8 points. When reaching the maximum level (20 points) of CHF prevention awareness, the expected adult mortality rate due to circulatory diseases will be 886.7 per 100,000 adult population.

**Table 2:** Characterization and analysis of the regression dependencies of mortality on the prevention awareness of patients.

Cause of death	Nosological form of circulatory system diseases	Pearson's correlation coefficient (p<0.05)	Share of factors accounted for by the regression model, %	Reduction in the mortality rate per 100,000 adult population with the patient awareness raised by 1 point
**Stroke**	Arterial hypertension	0.465	21.7	31.27
**Acute myocardial infarction**	Angina pectoris	0.411	16.9	7.05
**Circulatory system diseases**	Chronic heart failure	0.405	16.4	18.7

## DISCUSSION

The study results showed that patients’ awareness of the prevention of circulatory system diseases results from developed and implemented educational programs at Health Schools. The effectiveness of Health Schools for patients with AH, SAP and CHF was proven, and the need for further educational patient-specific care taking into account the existing individual risk factors was demonstrated. At the same time, activities of the primary healthcare sector are increasingly focused on prevention, confirming the possibility and effectiveness of health development throughout the entire life cycle. Based on changes in the patients’ health status, different levels of training programs for patients with circulatory system diseases are selected, and the patients are surveyed before and after the training. Taking into account the patients’ individual risk factors, the implemented computer program calculates a 10-year risk for fatal cardiovascular disease according to the Systematic COronary Risk Evaluation (SCORE) scale and the patient’s vascular age. Depending on individual risk factors, physicians create patient-specific prevention programs for each patient, which are uploaded online by the system to the patients’ portal and are displayed in their personal profiles. These programs are subjected to systematic editing by primary care physicians who support patients with circulatory system diseases taking into account individual risk factors.

We have picked up study programs for heart disease patients with pooling before and after studying. A medical specialist formed personalized prevention programs for each patient with individual risk factors. These programs have been edited by doctors of the first group who take care of patients with heart diseases and change personal risk factors.

Further improvement on these programs can be achieved through the implementation of positive international experiences with similar health promotion programs (for example, “All Kids Count Program”) [18]. In addition, studies show that almost 40% of deaths in the adult population could be accounted for the general negative behavioral patterns that could be preventatively modified through simple interventions and health promotion and education [19]. Therefore, it is crucial to continue developing health education in Tatarstan as its positive effect is scientifically observable and can be considered positive. Therefore, increasing the awareness range in AH prevention with a point will decrease the death rate because of stroke in 31.27 cases per 100,000 citizens (rxy=0.465). Knowledge about angina pectoris prevention will decrease the acute myocardial infarction death rate in 7.05 cases (rxy=0.411). Moreover, increasing knowledge about CHD prevention will decrease the acute myocardial circulatory system disease death rate in 18.7 cases per 100,000 citizens (rxy=0.405).

After studying at the Health School, the awareness increased to 15.13 points for AG, to 15.64 points for SAP, and to 14.71 points for CHD (p<0.001) compared to the control group (without any studying) – p<0.05. Furthermore, at the ending of the observation period, the awareness range reliably increased in the general group compared to the control group (p<0.001).

We observed a reliably lower frequency of circulatory system disease complications for the Health School students compared to the control group – 11.1 and 24.8 %, respectively (p<0.001). For acute myocardial infarction, these marks were 8.5 and 18.8 % (p<0.001) and 2.3 and 5.9% (p>0.05) for stroke.

## CONCLUSIONS

Extrapolation of the data on Tatarstan’s patient population was obtained through this study, taking into account the health of the Health Schools students, obtaining the regression equations of population mortality and the effects of training on it. The effectiveness of Health Schools for patients with cardiovascular pathology has been proven.

However, additional efforts are required to involve a wider range of patients and increase learning effectiveness to critical levels of awareness by introducing new forms of education in Health Schools since it statistically significantly increased the awareness level regarding disease nature and preventive measures. It is crucial to continue developing health education in Tatarstan as its positive effect is scientifically observable and can be considered positive.
